# Probing the *In Vitro* Cytotoxicity of the Veterinary Drug Oxytetracycline

**DOI:** 10.1371/journal.pone.0102334

**Published:** 2014-07-14

**Authors:** Zhenxing Chi, Rutao Liu, Hong You, Shanshan Ma, Hao Cui, Qiang Zhang

**Affiliations:** 1 State Key Laboratory of Urban Water Resource and Environment, Harbin Institute of Technology, Harbin, PR China; 2 School of Marine Science and Technology, Harbin Institute of Technology at Weihai, Weihai, PR China; 3 School of Environmental Science and Engineering, Shandong University, Jinan, PR China; University of Arkansas for Medical Sciences; College of Pharmacy, United States of America

## Abstract

The study investigated the effect of oxytetracycline (OTC) on the anti-oxidative defense system, the structure (hemolysis rate and morphology) and function (ATP enzyme activity) of human red blood cells (hRBCs) to investigate the possible toxic mechanism of OTC to hRBCs. The experimental results indicate that OTC can cause a decline in the function of the antioxidant defense system of hRBCs, resulting in oxidative stress. OTC can bring about morphological changes to hRBCs, and further leads to hemolysis, when the concentration of OTC is over 8×10^−5^ M (about 164 µg/ml). At a low OTC concentration, below 4×10^−5^ M (82 µg/ml), OTC can enhance the activity of ATP enzyme of hRBCs, known as hormesis. However, at a high concentration, above 4×10^−5^ M (about 82 µg/ml), the ATP enzymatic activity was inhibited, affecting the function of hRBCs. The estalished mechanism of toxicity of OTC to hRBCs can facilitate a deeper understanding of the toxicity of OTC in vivo.

## Introduction

**Figure 1:**
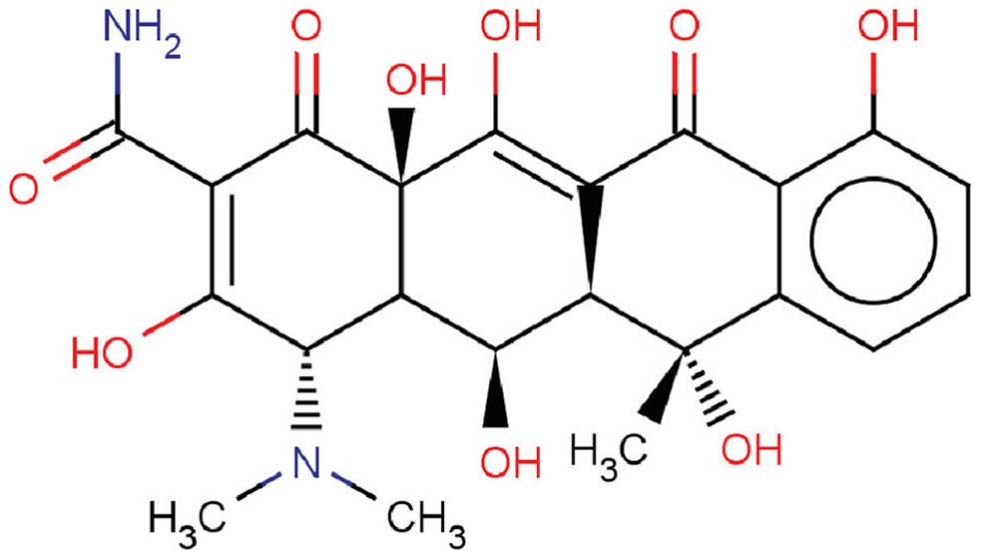
Molecular structure of OTC.

Oxytetracycline (OTC, structure shown in Fig. 1), a type of tetracycline antibiotics with broad-spectrum antibiotic activity [1], [2], is extensively applied for therapeutic purposes in humans as well as an antibiotic and growth promoter in intensive farming systems and aquaculture [3]. Effective therapy should be obtained when plasma OTC concentrations are above 4 µg/ml declared by the National Committee for Clinical Laboratory Standards (NCCLS) to achieve that the target bacteria are not resistant to OTC [4]. At the therapy dose of 20 mg/kg, the maximum serum concentration of OTC can reach 4.10 µg/ml for calves [5]. For different animals, the peak concentration and terminal half-life of OTC in blood can be achieved 4∼16 hours and 21∼63 hours after administration, respectively [6]–[10]. OTC concentrations in plasma can be kept at or above 0.5 µg/ml (minimum inhibitory concentration) for approximately 6 days [11].

The bioavailability of OTC is low [3], so the ingested OTC in animal body is metabolized partially and the residual OTC is excreted and released into soils, surface water and groundwater [3], [12]. OTC has been detected at nanogram to low-microgram per liter levels in wastewater effluents and natural waters [13]. OTC can bioaccumulate in various organisms including invertebrates and fish in food chain and enter human bodies by water drinking and food intake such as milk, meat and eggs (at nanogram to microgram per liter levels) [14]–[16], posing a threat to human health [13]. OTC can also be taken up by blood cells [17]. The Joint FAO/WHO Expert Committee of Food Additives and Contaminants (JECFA), at its 50th Meeting in 1998, established a group acceptable daily intake (ADI) of 0∼0.03 mg kg^−1^ body weight for the tetracyclines (oxytetracycline (OTC), tetracycline (TC) and chlortetracycline (CTC)), alone or in combination. The committee also recommended maximum residue limits (MRLs) of 100 µg L^−1^ in milk and muscle of all food-producing species [18], [19].

The toxicity of OTC residues in the environment including animal food, soils, surface and ground water, has attracted widespread attention [1], [2], [20], [21]. OTC can inhibit the antibody levels in fish (dose rate equivalent to 75 mg/kg body weight/day) [22], induces DNA damage in carp kidney cells (exposure dose 250 mg/l) [23], has teratogenic effects on carp embryos (exposure dose 127.5∼364.1 mg/l) [24], interacts with cytoplasmic protein synthesis (incubation with 100∼500 µg/ml of OTC) [25], and induce blood disorder in Juvenile Nile Tilapia *Oreochromis niloticus* (fish fed with 1.25% OTC and above) [26]. OTC also has effect on the secretion kinetics of the rat exocrine pancreas and can lead to the decrease in trypsin level in male wistar rats (OTC dose 30 mg/kg/d) [27], [28].

Red blood cells (RBCs), also referred to as erythrocytes, are the most abundant cells in the bloodstream [29]. The primary function of RBCs is to transport oxygen from the lungs to various parts of the body [30]. In addition, RBCs are also a key player in getting waste carbon dioxide from the tissues to the lungs of the body where it is expelled [30], and regulate blood pH [31]. However, intake of hazardous substances may result in injury of RBCs, affecting its functions.

OTC can cause significant reductions in several blood parameters including erythrocyte, hematocrit, and hemoglobin values, for Juvenile Nile Tilapia *Oreochromis niloticus* [26]. However, the effect of OTC on the anti-oxidative defense system of RBCs is still unknown. The changes in the activities of anti-oxidative defense system have been regarded as one of the toxic mechanisms of many hazardous substances. We also have little knowledge about the influence of OTC on the structure and function of RBCs. In this work, we investigated the toxicity indexes of OTC to human red blood cells (hRBCs) including the antioxidant capacity, the hemolysis rate and morphology (structure), and the ATP enzyme activity (function). We consider the work as a worst case scenario in view of the common concentration of OTC residues in the environment. The study is helpful for understanding the effect of OTC on hRBCs during the blood transportation process and its toxicity in vivo.

## Materials and Methods

### 2.1. Reagents and apparatus

EDTA dipotassium salt dihydrate (Tianjin Kermel Chemical Reagent Co., Ltd.) stabilized human blood samples were freshly obtained from Hospital of Shandong University (Jinan).

A stock solution of OTC (1.0×10^−3^ M) was prepared by dissolving 0.0497 g oxytetracycline hydrochloride (Sigma) in 100 ml of saline solution.

2,3-naphthalenedicarboxaldehyde (NDA) was obtained from Nippon Kasei Chemical Co., Ltd. Glutaraldehyde (Tianjin Kermel Chemical Reagent Co., Ltd.) was used as fixative. Isoamyl acetate was obtained from Tianjin Chemagent Research Co., Ltd. PBS buffer (10x, pH 7.4) was purchased from Shanghai Biocolor BioScience & Technology Company. All other reagents were of analytical grade.

All UV-visible absorption spectra and absorption value were measured on a UV-2450 spectrophotometer (SHIMADZU, Kyoto, Japan). Automatic balance centrifuge (LDZ4-2, Jiangsu Jintan Medical Instrument Factory) was used for centrifugation. Vortex mixer (vortex-6) was purchased from Kylin-Bell Lab Instruments Co., Ltd. Digital dry bath incubator (HB-100, Hangzhou Bioer Technology Co., Ltd) was used to control temperature of the samples.

### 2.2. Ethics statement

The study was approved by the Ethics Committee of Hospital of Shandong University (Jinan). Written informed consent was obtained from all study participants.

### 2.3. Determination of the activities of SOD, CAT and GSH-Px

SOD is a potent protective enzyme that can selectively scavenge O_2_^.−^ by catalyzing its dismutation to H_2_O_2_ and molecular oxygen (O_2_) to protect the cells from being injured [32]. CAT catalyzes the degradation of H_2_O_2_ to H_2_O and O_2_: 2H_2_O_2_→2H_2_O+O_2_ [33]. The GSH-Px is an important enzyme extensively existing in vivo, which can specifically catalyze the reduction of H_2_O_2_ by GSH to protect the integrity of the structure and function of the membrane. With the activities of SOD, CAT and GSH-Px as the indicators, we studied the effect of OTC on the antioxidant defense of hRBCs.

#### 2.3.1. SOD activity determination

Test principle: superoxide anion radical (O_2_^.−^), produced in xanthine and xanthine oxidase reaction systems, can oxidize hydroxylamine to nitrite, which shows violet under the effect of color reagent. The absorbance can be measured with visible or UV-vis spectrophotometer. When the measured sample contains superoxide dismutase (SOD), its specific inhibition of O_2_^.−^ can reduce the formation of nitrite, the absorbance value is lower than that of the control with colorimetry. The SOD activity was determined by utilizing the SOD detection kit (Nanjing Jiancheng Bioengineering Institute).

The freshly obtained blood sample (1 ml) was added to 2 ml of PBS, mixed by vortexing, and then the hRBCs were isolated from serum by centrifugation at 2000 rpm for 5 min. After being washed two times with 2 ml of PBS solution, the purified hRBCs were diluted to 2 ml with PBS. 0.2 ml of the diluted cell suspension was added to 0.8 ml OTC solutions of different concentrations and mixed by vortexing, then incubated for 3 hours under gentle shaking. Following incubation, the samples were centrifuged (2000 rpm×5 min), and the supernatant was discarded and the pellet was resuspended in 1 ml PBS. The SOD activity of the sample was measured according to the procedure of the detection kit. The relative SOD activity was calculated using the following formula [34]:
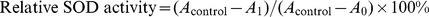



Where *A*_control_ is the absorbance of the control tube, *A*_1_ and *A*_0_ are the absorbances of the testing tube of RBCs with and without OTC, respectively.

#### 2.3.2. CAT activity determination

Test principle: the decomposition of hydrogen peroxide (H_2_O_2_) by catalase (CAT) can be rapidly stopped by ammonium molybdate. The residual H_2_O_2_ interacts with ammonium molybdate to produce a yellowish complex. The amount formed can be determined at 405 nm to calculate the CAT activity.

The freshly obtained blood sample (1 ml) was washed three times with 2 ml PBS by centrifugation for 5 min at 2000 rpm. The purified hRBCs were diluted to 10 ml with PBS. 0.2 ml of the diluted cell suspension was mixed with 0.8 ml OTC solutions of different concentrations by vortexing, and then incubated for 3 hours under gentle shaking. Following incubation, the samples were centrifuged (2000 rpm×5 min), and the supernatant was discarded and the pellet was resuspended in 0.3 ml ultrapure water to achieve hemolysis. The hemolytic blood was used to measure the CAT activity according to the procedure of the detection kit (Nanjing Jiancheng Bioengineering Institute).

#### 2.3.3. GSH-Px activity determination

Test principle: glutathione peroxidase (GSH-Px) can promote the reaction of H_2_O_2_ with reduced glutathione (GSH) to produce H_2_O and GSSG. The GSH-Px activity can be expressed by the speed of the enzymatic reaction, detected by the consumption of GSH. In the experiment, the GSH-Px activity was determined by utilizing the GSH-Px detection kit (Nanjing Jiancheng Bioengineering Institute) with spectrophotometry method.

The freshly obtained blood sample (1 ml) was washed, diluted, incubated with OTC and centrifuged according to the procedure in Section 2.3.2. The supernatant was discarded and the pellet was resuspended in 1 ml ultrapure water to achieve hemolysis. 0.2 ml of the hemolytic blood was used to measure the GSH-Px activity according to the procedure of the detection kit (Nanjing Jiancheng Bioengineering Institute).

### 2.4. Determination of GSH

Test principle: GSH is an important nonenzymatic antioxidant. It participates in the maintaining of redox equilibrium which may alleviate cellular oxidative injury. In this work, NDA was used to label the intracellular GSH of hRBCs. Nonfluorescent NDA can readily penetrate the cell membrane, interacting with the nonfluorescent GSH to produce a green fluorescent GSH-NDA derivative [35]. The fluorescence intensity of GSH-NDA derivative was directly proportional to the intracellular content of GSH by fluorescence image analysis. NDA can react concomitantly with the amino and thiol groups of GSH, and no additional nucleophile reagent is required [36]. So the bifunctional reaction is rapid, nonenzymatic, and highly selective [36].

The freshly obtained blood sample (1 ml) was washed, diluted, incubated with OTC and centrifuged according to the procedure in Section 2.3.2. The supernatant was discarded and the pellet was resuspended in 1 ml PBS. For the determination of GSH, 0.3 ml cell suspension was incubated with 100 µl 0.02 mol/L NDA solution and 0.6 ml PBS for 0.5 hour in the dark at room temperature. 0.05 ml of the suspension was placed on the no-clean cover glass for investigation (24×50 mm, Citotest Labware Manufacturing Co., Ltd).

An inverted microscope (Model IX81, Olympus, Tokyo, Japan) equipped with a 10×objective (PlanApo, Olympus, Tokyo, Japan), a mercury lamp, a mirror unit consisting of 470–490 nm excitation filter (BP470–490), a 505 nm dichromatic mirror (DM 505), a 510–550 nm emission filter (BA510–550) and a 16-bit thermoelectrically cooled EMCCD (Cascade 512B, Tucson, AZ, USA) were used for epifluorescence measurements. The derivatized GSH was excited with a 470–490 nm light ray through the objective. The fluorescence emitted by these molecules was collected by the same objective and the fluorescence images were acquired by the EMCCD. Image acquisition was controlled by the MetaMorph software (Universal Imaging, Downingtown, PA, USA). The micrographs of hRBCs without derivatization were observed by the inverted microscope under bright field and epifluorescence illumination conditions.

### 2.5. Determination of Malondialdehide (MDA)

Test principle: MDA is the degradation product of lipid peroxidation which can indicate the level of oxidative stress. It can condense with TBA to form a coloured MDA-TBA complex, with the maximum absorption peak at 532 nm that can be measured by visible absorption spectrophotometry.

The freshly obtained blood sample (1 ml) was washed, diluted, incubated with OTC and centrifuged according to the procedure in Section 2.3.1. The supernatant was discarded and the pellet was resuspended in 1 ml ultrapure water to achieve hemolysis. 0.1 ml of the hemolytic blood was used to measure the MDA concentration according to the procedure of the detection kit (Nanjing Jiancheng Bioengineering Institute).

### 2.6. Hemolysis assay

The freshly obtained blood sample (1 ml) was washed, diluted and mixed with OTC according to the procedure in Section 2.3.2. RBC incubation with ultrapure water and PBS were used as the positive and negative controls, respectively. All the sample tubes were kept in static condition at room temperature for 3 h. Finally, the mixtures were centrifuged at 2000 rpm for 5 min, and 500 µl of supernatant of all samples was diluted with 2.5 ml ultrapure water. The absorbance values of the diluted supernatants (3 ml) at 540 nm were determined by the UV-2450 spectrophotometer [37]. The hemolysis rate of hRBCs was calculated using the following formula [38]: 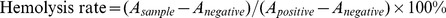



Where *A*_sample_, *A*_positive_ and *A*_negative_ are the absorbances of sample, the positive and negative controls, respectively.

### 2.7. Scanning electron microscopy (SEM) studies of hRBCs

The freshly obtained blood sample (1 ml) was washed according to the procedure in Section 2.3.1. The purified hRBCs were diluted by 25 times with PBS. 0.2 ml of the diluted cell suspension was incubated with 0.8 ml OTC solutions of different concentrations for 3 hours under gentle shaking. Following incubation, the samples were centrifuged (2000 rpm×5 min) and the supernatant was discarded. Fixation was performed by addition of 2.5% glutaraldehyde and 12 h incubation. The fixed samples were washed with 0.1 M phosphate buffer for more than 3 h. Then, the sample was fixed in osmium tetroxide (1%) for 1∼1.5 h and washed in double-distilled water for 2 h. Dehydration was done with increasing concentrations of ethanol (50%, 70%, 80%, 90% and 100%) twice. The ethanol was displaced by isoamyl acetate (100%) for 15 min (twice). Finally, the sample was dried with the conventional critical point drying method, platinum-coated with ion sputtering coater (Eiko, IB-5) and then observed with a scanning electron microscope (Hitachi, S-570) to investigate the effect of OTC on the morphology of hRBCs.

### 2.8. ATPase activity assay determination

ATPase is an important enzyme existing in the membrane of cells in vivo [39]. It functions in maintaining ionic and osmotic balance inside and outside the cell, maintaining transmembrane electrochemical gradients, and in cellular energy metabolism [39], [40]. The reduction in ATPase activity can result in the disorder of the functions, which affects the normal function of cells [39], [40]. For example, a rise in internal Na^+^, K^+^ or Ca^2+^, Mg^2+^ ions of RBCs can cause changes in cell shape and volume, increase in cellular rigidity and hemolysis. ATPase can cause the decomposition of ATP into ADP and inorganic phosphorus. The activity of ATPase can be determined by measuring the amount of inorganic phosphorus.

The freshly obtained blood sample (1 ml) was washed with PBS, incubated with OTC, and then centrifuged according to the procedure in Section 2.3.1. The supernatant was discarded and the pellet was resuspended in 0.5 ml ultrapure water to achieve hemolysis. 0.2 ml of the hemolytic blood was used to measure the ATPase activity according to the procedure of the detection kit (Nanjing Jiancheng Bioengineering Institute).

### 2.9. Statistical analysis

Data are from three independent experiments and presented as mean ± SD in the study of the effect of OTC on the activities of SOD, CAT, GSH-Px and ATPase, content of GSH and MDA, and hemolysis rate of hRBCs.

## Results and Discussion

### 3.1. Effects of OTC on antioxidant capacity of hRBCs

Radicals are present in tissues in vivo as free and bound forms. The bound radical is necessary for normal physiological activity. However, the free radical is highly reactive, easily combining with electrons from tissue macromolecules to achieve more stability through electron pairing. In toxicology, there is interest in free radicals and reactive oxygen species (ROS), which can be produced by the normal metabolism of cells or by the exogenous factors (e.g. smoking, radiation, dust). ROS are free radicals and non-free radical oxygen-containing molecules that have higher chemical reactivity than ground state molecular oxygen [41], including species such as O_2_^.−^, hydroxyl radicals (·OH), nitric oxide (NO·), singlet oxygen (^1^O_2_) and H_2_O_2_ [32]. ROS, within physiological concentrations, play an important role in regulating the body's normal physiological functions such as apoptosis, gene expression and signal transduction [42]. However, when free radicals and antioxidative processes are not balanced, that is, when the production of ROS exceeds the scavenge ability of the body's defense system, or the damaged body's defense system can not function properly, free radicals can produce oxidative stress, inducing the oxidation of biological macromolecules (nucleic acids, proteins, lipids et al), damage to the structure and function of cells, and diseases such as pulmonary fibrosis, epilepsy, hypertension and atherosclerosis [43], [44]. Antioxidants, being mainly natural molecules, can prevent the uncontrolled formation of ROS or inhibit reactions of ROS with biological structures. Enzymes such as SOD, CAT and GSH-Px provide the main antioxidant defense [45]. There must be a balance between the three enzymes to remove ROS from the body properly [46]. As non-enzymatic defense, the role of dietary supplements such as GSH, vitamins (C, E) and carotenoids, is also very important to control oxidative injury [33], [47]. In this section, we studied the effect of OTC on the antioxidant defense of hRBCs including SOD, CAT, GSH-Px and GSH.

**Figure 2:**
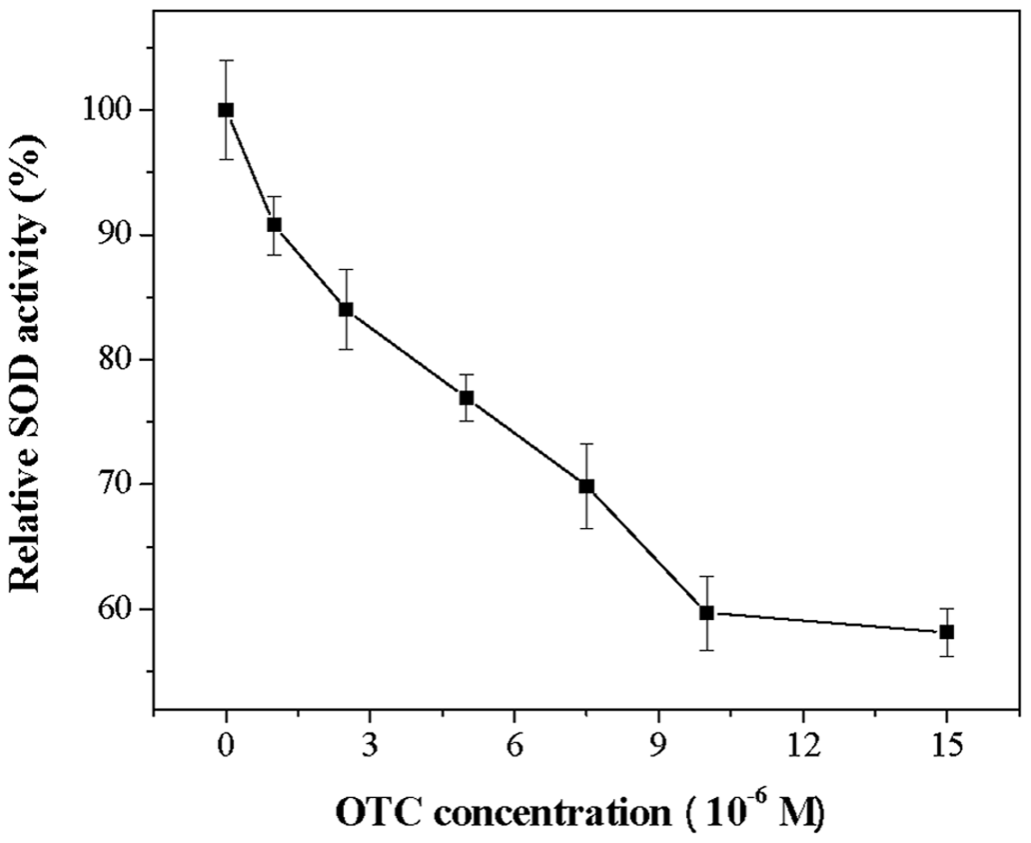
Effect of OTC on SOD activity of hRBCs. Data represent the mean ± SD of three independent experiments.

**Figure 3:**
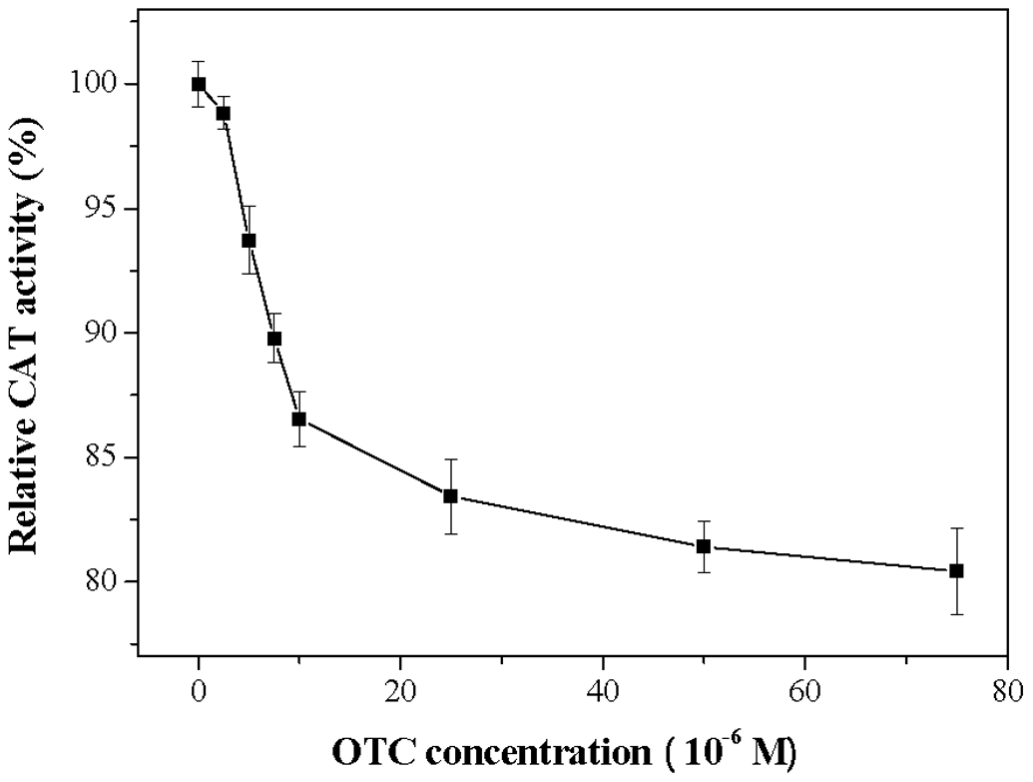
Effect of OTC on CAT activity of hRBCs. Data represent the mean ± SD of three independent experiments.

**Figure 4:**
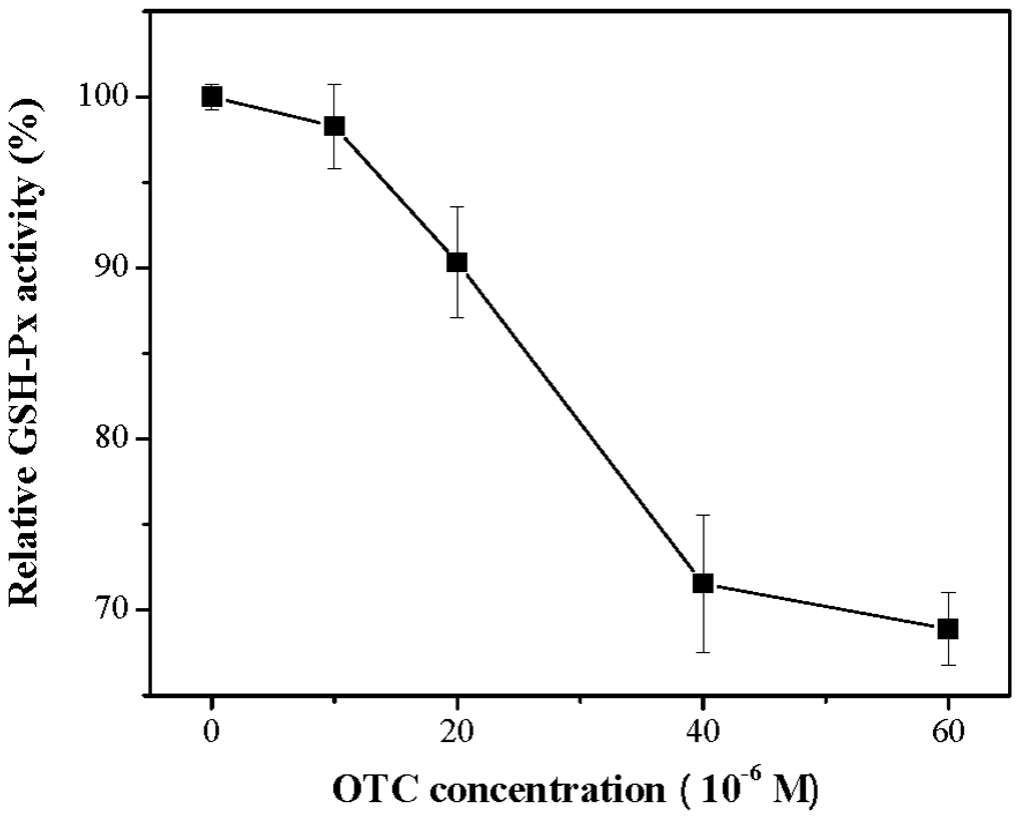
Effect of OTC on GSH-Px activity of hRBCs. Data represent the mean ± SD of three independent experiments.

In the experiments, SOD activities decreased with increasing OTC concentrations ranging from 0 to 1.5×10^−5^ M and decreases by 41.85% at OTC concentration 1.5×10^−5^ M (Fig. 2). CAT activity also decreased as OTC concentration increased from 0 to 7.5×10^−5^ M. 19.58% reduction in CAT activity was observed at OTC concentration 7.5×10^−5^ M (Fig. 3). For GSH-Px, the activity also deceased at increasing OTC concentrations (0–6×10^−5^ M) (Fig. 4), and was only 68.89% of the initial concentration when exposed to 6×10^−5^ M OTC.

**Figure 5:**
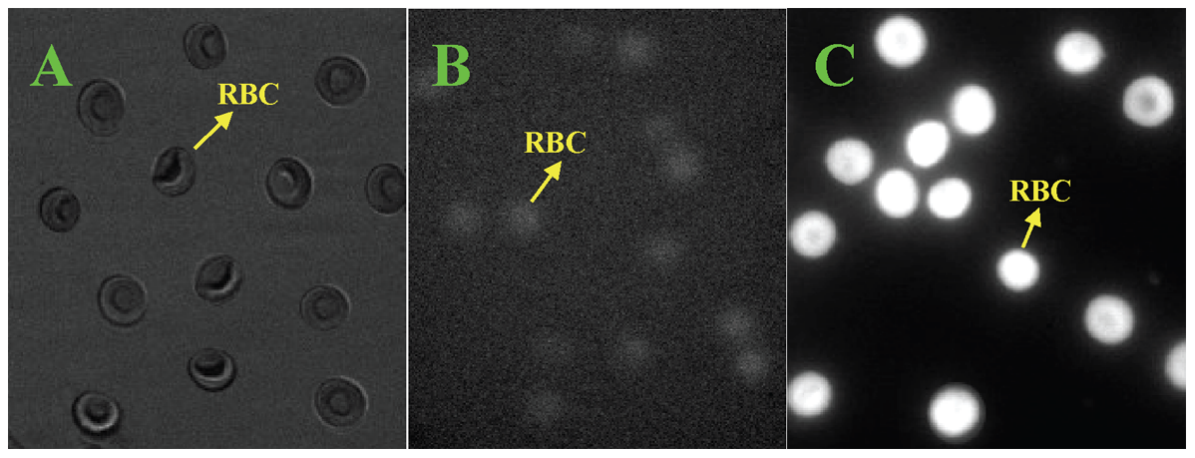
Optical and fluorescence images of hRBCs at 60× magnification (GSH derivatized by NDA). A and B are the hRBCs under bright field and epifluorescence illumination condition, respectively. C is the derived hRBCs under epifluorescence illumination condition.

**Figure 6:**
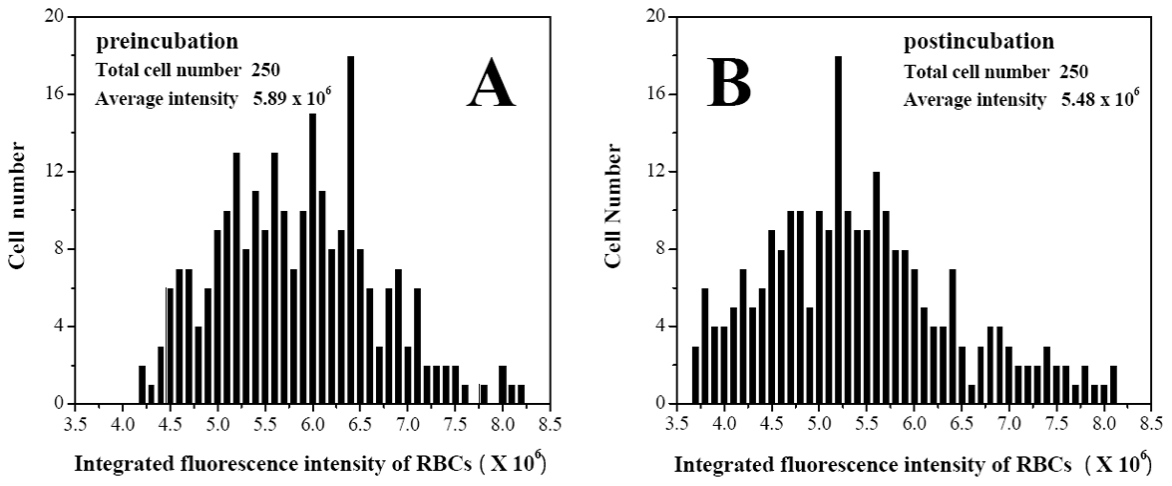
The distribution map of integrated fluorescence intensity (representing glutathione content) of hRBCs. A, hRBCs only; B, hRBCs incubating with 6×10^−5^ mol L^−1^ OTC.

**Figure 7:**
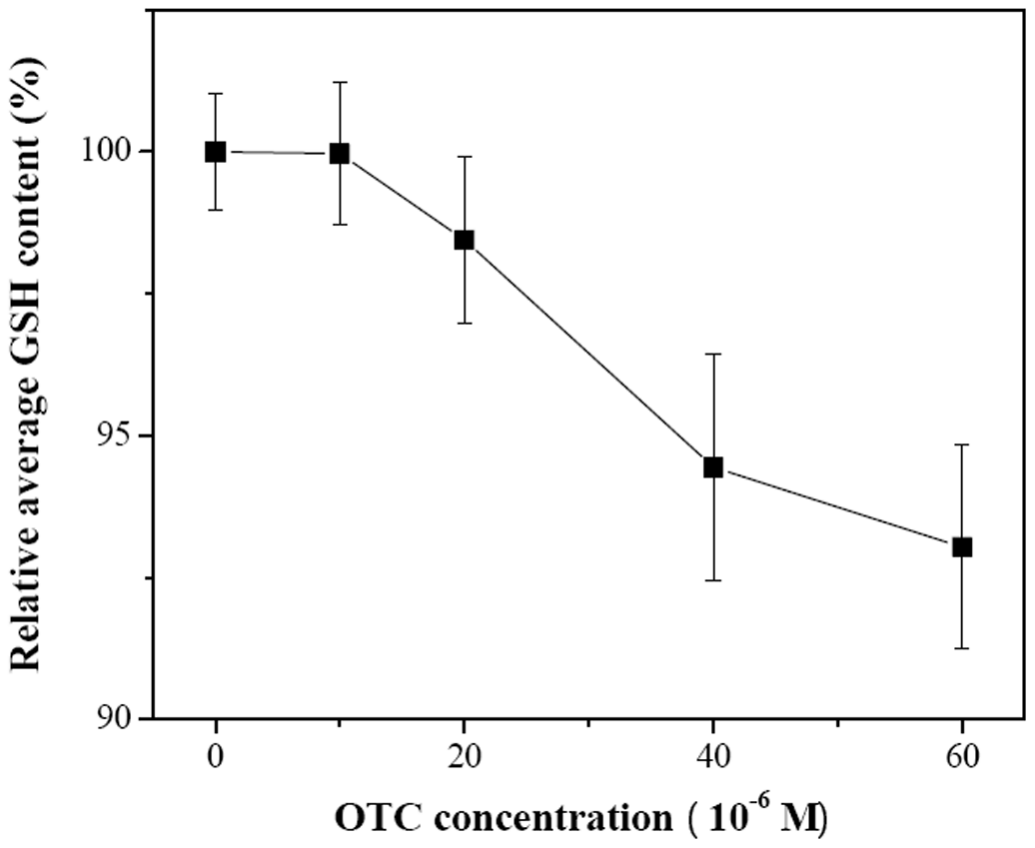
Effect of OTC on GSH content of hRBCs. Data represent the mean ± SD of three independent experiments.

NDA was used to probe the content of GSH in hRBCs. Fig. 5A shows the hRBCs under bright field. When observed under epifluorescence illumination condition, the cell without derivation with NDA had a very weak fluorescence (Fig. 5B). However, the derived hRBCs exhibited a strong fluorescence (Fig. 5C). The integrated fluorescence intensity of the hRBCs in Fig. 5B is only about 0.6%∼0.7% of that of the derived hRBCs in Fig. 5C, so it is negligible. Therefore, the GSH content can be represented by the integrated fluorescence intensity of the derived hRBCs (Fig. 5C). To investigate the influence of OTC on the GSH concentration in hRBCs, 250 hRBCs were selected for every different OTC concentration used. Fig. 6 shows the distribution map of integrated fluorescence intensity. With increasing OTC concentration, the GSH concentration decreased (Fig. 7). At OTC concentration 6×10^−5^ M, GSH content reduced to 93% of the initial concentration (5.48×10^6^ compared to 5.89×10^6^ average fluorescence intensity).

**Figure 8:**
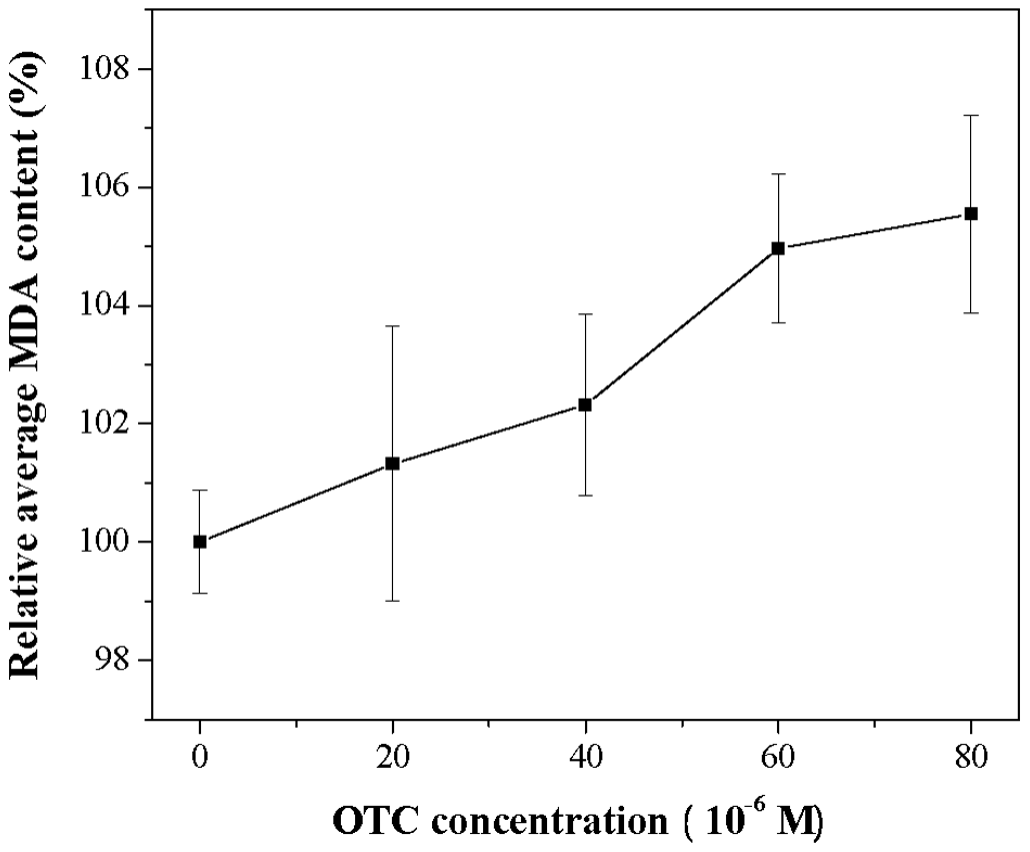
MDA content of hRBCs under different OTC concentrations. Data represent the mean ± SD of three independent experiments.

Exposure of hRBCs to OTC resulted in decreased antioxidant activity of enzymes including SOD, CAT and GSH-Px, and non-enzymatic defenses such as GSH, which all scavenge ROS [32]. So, OTC can reduce antioxidant capacity of hRBCs, which may predispose cells to oxidative injury [48]. To confirm this, we determined the effect of OTC on the concentration of MDA in hRBCs, which is a marker of lipid peroxidation induced by oxidative injury [49], [50]. From Fig. 8, we can seen that MDA content in hRBCs increased with increasing OTC concentrations ranging from 0 to 8×10^−5^ M. 5.5% increment in MDA content was observed at OTC concentration 8×10^−5^ M.

In conclusion, OTC can reduce the antioxidant capacity of hRBCs and induce lipid peroxidation, causing oxidative injury.

### 3.2. Impact of OTC on the hemolytic activity of hRBCs

**Figure 9:**
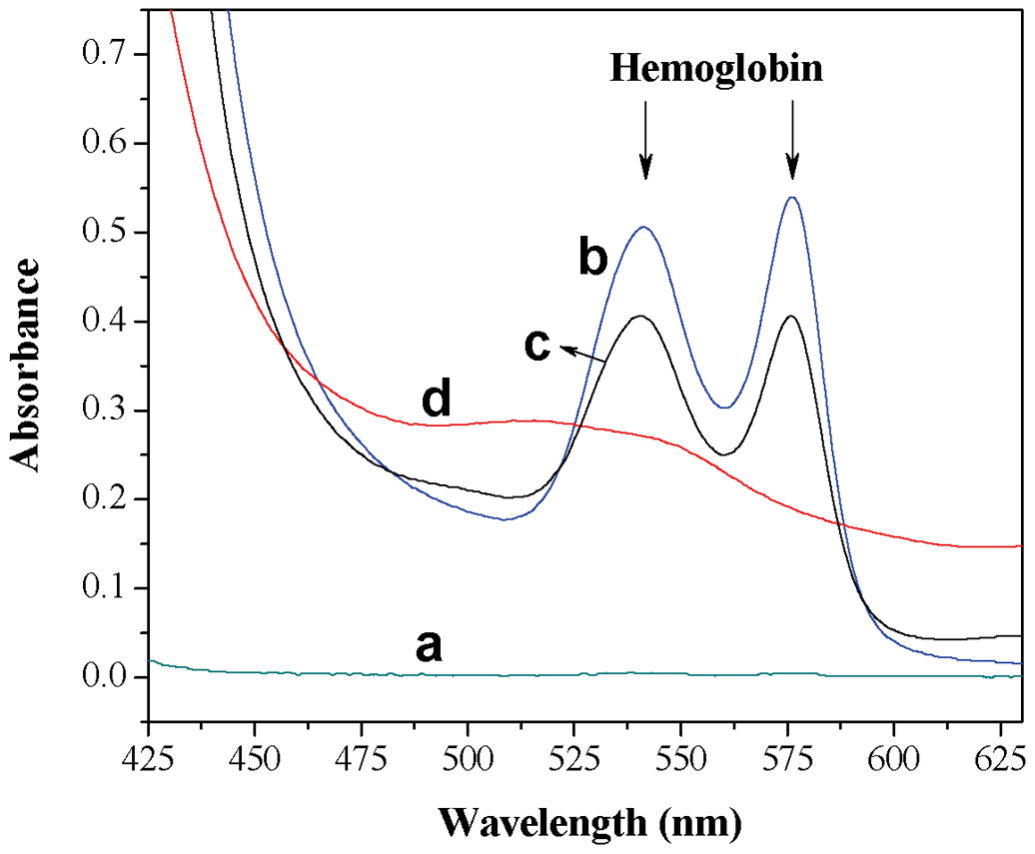
Absorption spectra. (a) supernatant for the hRBCs suspension after centrifugation; (b) hemolysate (incubated with ultrapure water); (c) supernatant for the hRBCs incubated with 1.1×10^−4^ mol/L OTC after centrifugation; (d) hemolysate incubated with 4×10^−4^ mol/L OTC. a, b, c and d have the same number of hRBCs obtained from the same blood sample.

In the process of lipid peroxidation in the cell membrane by ROS, membrane fluidity and permeability is altered, affecting cellular structure and function. When RBCs are destroyed, known as hemolysis, contents of the RBCs are released into the bloodstream and can accumulate, causing health problems that can occur suddenly at any time [51], [52], including kidney failure and serious blood clots, which may cause damage to important organs of the body such as the liver, brain, and lung [53]. So, it is very important to investigate the effect of toxic substances on the hemolytic activity of RBCs. The hemolysis caused by toxic substances, which can be expressed as the leakage of hemoglobin, is reflective of membrane damage to the RBCs [38]. After centrifugation, the supernatant from unhemolyzed hRBCs has no absorbance at the range of 425 to 625 nm (Fig. 9). However, the released hemoglobin from damaged hRBCs has two absorption peaks at about 540 nm and 576 nm (Fig. 9). In this research, the absorbance values at 540 nm were used to evaluate the hemolysis rate of hRBCs [37].

**Figure 10:**
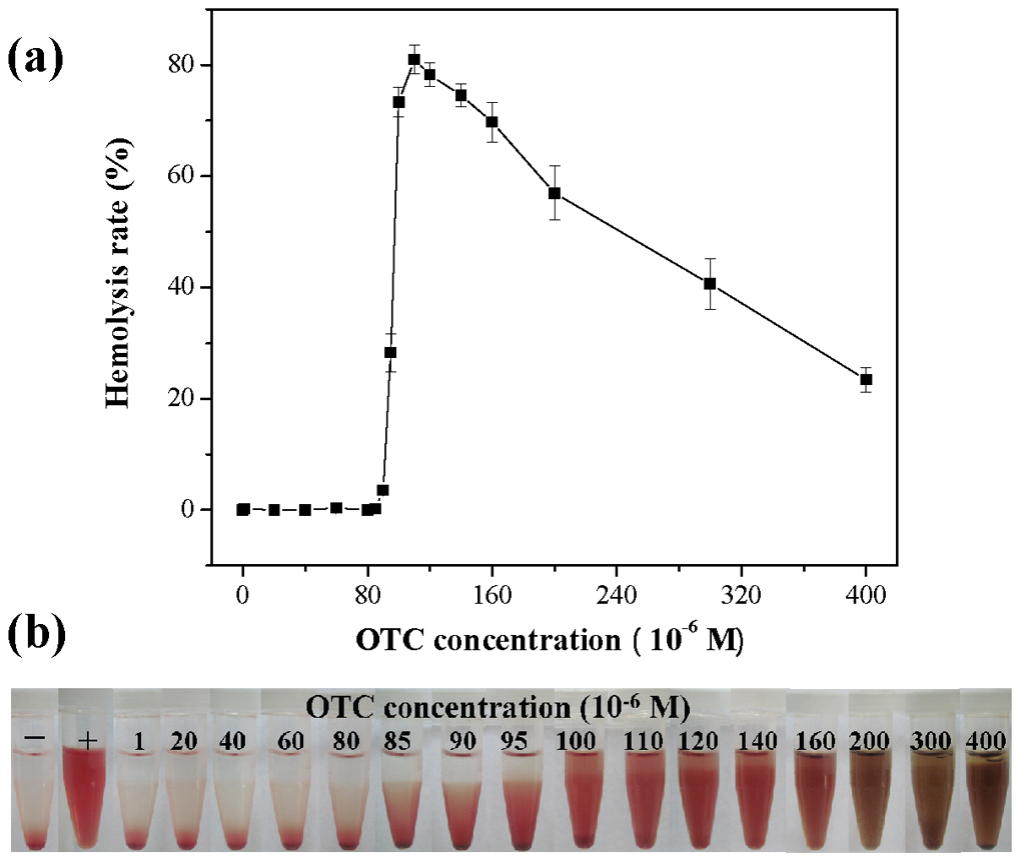
Hemolysis rate (a) and photographs of hemolysis (b) of hRBCs incubated with OTC. (a) hRBCs incubated with OTC at different concentrations ranging from 1 to 4×10^−4^ mol L^−1^ for 3 h. Data represent the mean ±SD from at least three independent experiments. (b) The presence of red hemoglobin in the supernatant indicates damaged hRBCs. D.I. water (+) and PBS (−) are used as positive and negative control, respectively.

To determine if OTC produces hemolysis of hRBCs, hRBCs were exposed to OTC solutions at concentrations ranging from 0 to 4×10^−4^ M for 3 h. The experimental result (Fig. 10a) showed that when the OTC concentration was lower than 8×10^−5^ M, OTC did not induce hemolysis and no hemoglobin was observed in the supernatant, as seen in Fig. 10b, indicating that hRBCs can preserve itself from hemolysis. Hemolysis of hRBCs increased significantly with increasing OTC concentrations starting at a concentration of 8×10^−5^ M, based on the increase in hemoglobin concentrations in the supernatant (Fig. 10b). When the OTC concentration was 1.1×10^−4^ M, hemolysis was 80.99%, however, at higher OTC concentrations (above 1.1×10^−4^ M), hemolysis declined with increasing OTC. The phenomenon was not caused by the oxidation of hemoglobin to methemoglobin, because the absorption peak of methemoglobin at 630–631 nm did not appear in the experimental result. According to our previous study [54], OTC can bind into the central cavity of the hemoglobin molecule. So, we think that OTC interacts with the released hemoglobin, causing the absorbance to decrease. The experimental result that the absorbance of the hemolysate at 540 nm declined after the addition of OTC verified our conclusion.

In conclusion, at lower OTC concentrations (≤8×10^−5^ M), hRBCs are protected from membrane hemolysis. OTC has hemolytic activity at a concentration greater than 8×10^−5^ M.

### 3.3. Effect of OTC on the morphology of hRBCs

**Figure 11:**
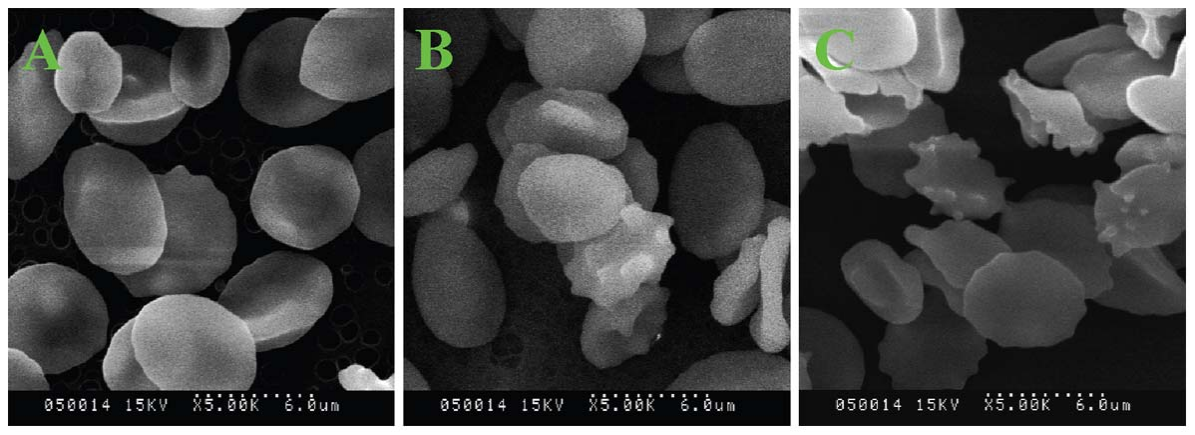
Scanning electron micrographs of hRBCs incubated in PBS containing OTC. The OTC concentration for A, B and C is 0, 1.0×10^−5^ mol L^−1^ and 5.0×10^−5^ mol L^−1^, respectively.

Under physiological conditions, the normal hRBCs have a biconcave discoid shape (discocyte) ∼8 µm in diameter, consisting of a lipid bilayer membrane filled with aqueous hemoglobin solution [55], [56]. The effect of OTC on hRBC morphology was investigated by SEM. At the concentration of 1.0×10^−5^ M, OTC produced spicule formation on the hRBC membrane (acanthocyte, Fig. 11B) compared with the control hRBCs (Fig. 11A). At a higher concentration of 5.0×10^−5^ M, hRBCs became more deformed, with more cytoplasmic projections (Fig. 11C). The morphological alterations of hRBCs can ultimately lead to the destruction of the cells [57], which explains the observed high hemolytic activity of OTC. According to the bilayer-couple hypothesis [58], shape changes arise from the intercalation of OTC in either the outer or the inner monolayer of the RBC membrane. The lipid bilayer is the main permeability barrier of membrane, so the structural perturbation induced by OTC will affect its permeability, and thus may affect the function of ion channels, receptors, and enzymes immersed in the membrane lipid [59].

### 3.4. Influence of OTC on the ATPase activity of hRBCs

ATPase is an important enzyme existing in the membrane of cells in vivo [39]. It functions in maintaining ionic and osmotic balance inside and outside the cell, maintaining transmembrane electrochemical gradients, and in cellular energy metabolism [39], [40].

**Figure 12:**
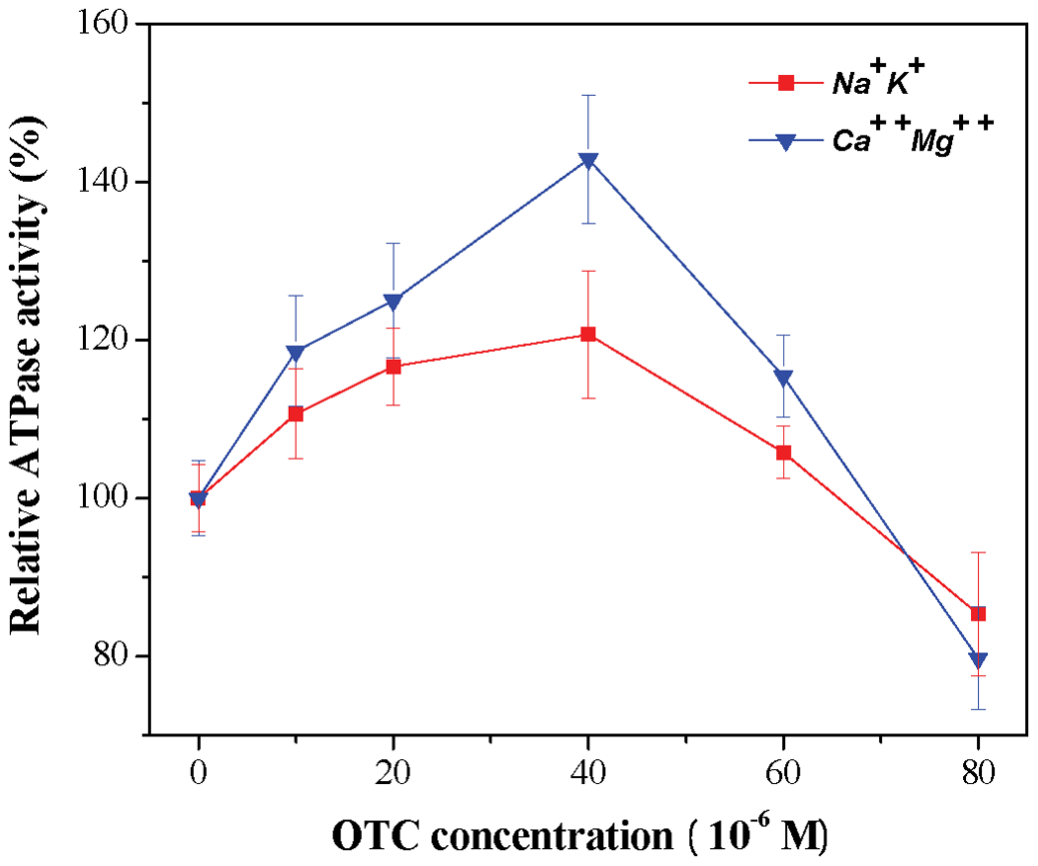
Effect of OTC on ATPase activity of hRBCs. Data represent the mean ± SD of three independent experiments.

In this section, we investigated the influence of OTC on the activity of (Na^+^, K^+^)-ATPase and (Ca^2+^, Mg^2+^)-ATPase. From Fig. 12, we found that both the activity of (Na^+^, K^+^)-ATPase and (Ca^2+^, Mg^2+^)-ATPase increased with increasing OTC concentrations ranging from 0–4×10^−5^ M. At an OTC concentration of 4×10^−5^ M, the activity increased to 120.67% and 142.86% of the initial level for (Na^+^, K^+^)-ATPase and (Ca^2+^, Mg^2+^)-ATPase, respectively. However, at higher OTC concentration, the activity decreased. Inhibition rates of 16.67% and 22.25% were observed at the OTC concentration of 4×10^−5^ M for the activity of (Na^+^, K^+^)-ATPase and (Ca^2+^, Mg^2+^)-ATPase, respectively.

In summary, At a low concentration of OTC, the ATPase activity increased, known as hormesis. However, at a higher concentration, OTC can inhibit the activity of ATPase, affecting cell function, which verifies the analysis of the experimental results of the antioxidant capacity determination and morphology investigation of hRBCs under different concentrations of OTC (3.1 and 3.3).

## Conclusions

The research systemically studied the effect of OTC on the anti-oxidative defense system, the structure (hemolysis rate and morphology) and function (ATP enzyme activity) of hRBCs. The experimental results indicated that OTC can cause a decline in the function of the antioxidant defense system of hRBCs, enhancing the lipid peroxidation, which further result in the change of morphology and ATP enzyme activity of hRBCs. The established possible toxic mechanism of OTC to hRBCs can facilitate a deeper understanding of the toxicity of OTC in vivo.
